# Life‐Threatening Amlodipine Overdose: A Rare Case of Noncardiogenic Pulmonary Edema and Respiratory Failure Requiring ICU Care

**DOI:** 10.1002/ccr3.71538

**Published:** 2025-11-29

**Authors:** Rohit Pandit, Pradeep Masuta, Nishchal Regmi, Anil Nepali, Gajendra Acharya, Muhammad Jibran, Heena Maharjan, Nimesh Shrestha

**Affiliations:** ^1^ Tidal Health Peninsula Regional Internal Medicine Salisbury USA; ^2^ Kist Medical College and Teaching Hospital Lalitpur Nepal; ^3^ Kathmandu University School of Medical Sciences Dhulikhel Nepal

**Keywords:** amlodipine overdose, calcium channel blocker toxicity, high‐dose insulin therapy, noncardiogenic pulmonary edema, respiratory failure

## Abstract

Amlodipine, a dihydropyridine calcium channel blocker (CCB), is commonly prescribed for hypertension and anginal chest pain. An overdose of this medication can result in life‐threatening complications such as vasodilatory shock and pulmonary edema. Noncardiogenic pulmonary edema in this setting is rare and not well understood. We report the case of a 57‐year‐old female with a complex medical history, including diffuse large B‐cell lymphoma (DLBCL) in remission, asthma, and depression, who presented after ingesting approximately 87 tablets (870 mg) of amlodipine in a suicide attempt. The patient developed distributive shock requiring vasopressor support and acute hypoxic respiratory failure due to noncardiogenic pulmonary edema requiring intubation, mechanical ventilation, and intensive care unit (ICU) admission. Her course was complicated by prolonged mechanical ventilation and ventilator‐associated pneumonia (VAP), acute kidney injury (AKI), and metabolic derangements. With aggressive supportive therapy—including vasopressors, diuresis, antibiotics, and mechanical ventilation—the patient made a full recovery. This case is notable because NCPE is an uncommon manifestation of amlodipine toxicity [5], and because the patient survived despite multiple comorbidities. Early recognition and multidisciplinary management are critical to survival.

## Summary

- Amlodipine overdose can cause life‐threatening noncardiogenic pulmonary edema and shock. Early recognition and aggressive ICU management—including high‐dose insulin therapy, vasopressors, and ventilatory support—are critical for survival. Clinicians should consider CCB toxicity in unexplained hypotension with hyperglycemia. NCPE is uncommon but documented in amlodipine toxicity [5], emphasizing the need for early recognition.

## 1 Introduction

Calcium channel blocker (CCB) toxicity is a potentially fatal condition that requires prompt identification and aggressive management. CCBs are classified into two broad categories: non‐dihydropyridines and dihydropyridines. The latter, including amlodipine, predominantly cause peripheral vasodilation and are less likely to affect myocardial conduction and contractility than non‐dihydropyridines such as verapamil or diltiazem [1, 2]. Amlodipine has a prolonged half‐life and a high affinity for vascular smooth muscle, which makes overdose particularly dangerous due to the sustained vasodilatory effects that can lead to significant hypotension and shock [3].

While CCB overdose is mostly characterized by hypotension and bradycardia, complications like pulmonary edema, arrhythmias, and even multi‐organ failure can also occur [4]. Noncardiogenic pulmonary edema, in particular, is an uncommon but serious manifestation that can mimic acute respiratory distress syndrome (ARDS) in critically ill patients [5]. The pathophysiology behind noncardiogenic pulmonary edema in amlodipine overdose remains unclear, but it is thought to result from increased pulmonary capillary pressure and endothelial dysfunction due to vasodilation [6]. In this case report, we describe a severe overdose of amlodipine leading to profound vasodilatory shock, respiratory failure, and noncardiogenic pulmonary edema requiring ICU admission, mechanical ventilation, and a prolonged recovery period.

Additionally, this case is clinically important because NCPE is uncommon but documented [5], and survival after massive ingestion in a patient with multiple comorbidities highlights the importance of timely ICU care.

## 2 Case Report

### 2.1 Clinical History and Examination

A 57‐year‐old woman with asthma, thoracic aortic aneurysm, diverticulitis, DLBCL in remission, major depressive disorder, and a 41‐pack‐year smoking history presented after ingesting 85–87 tablets of 10 mg amlodipine. She also sustained superficial wrist lacerations. Fluoxetine had recently been initiated. On arrival, BP was 82/48 mmHg and HR 112 bpm. *β*‐blocker overdose was initially considered during undifferentiated shock but was quickly ruled out because of sinus tachycardia, negative co‐ingestant screens, and confirmed massive amlodipine ingestion [2, 7].

### 2.2 Differential Diagnosis, Investigations, and Treatment

**FIGURE 1:**
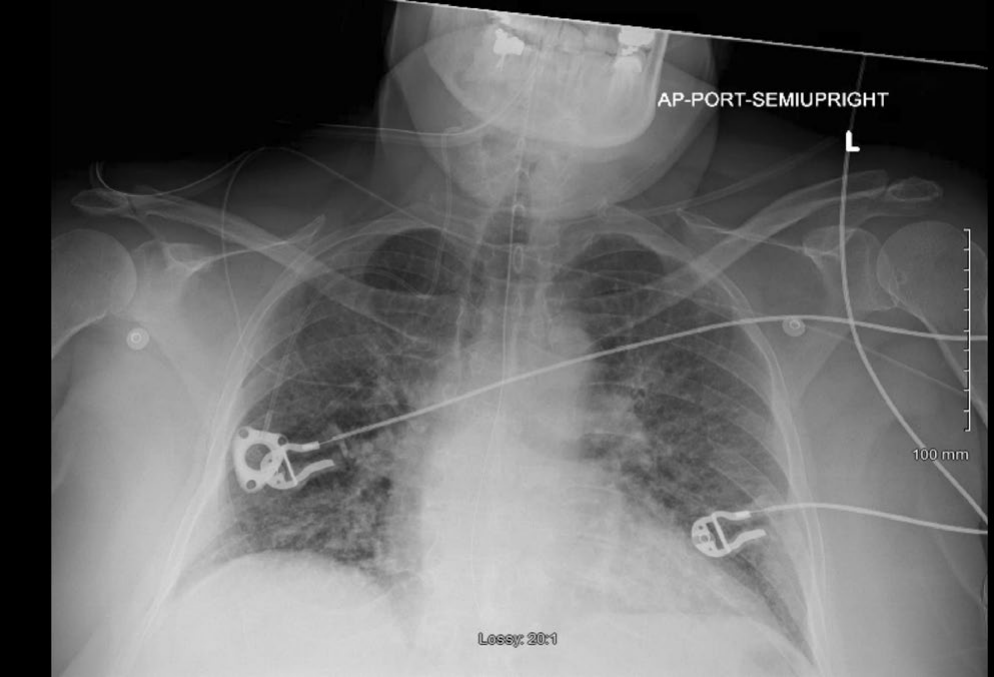
Presenting chest X‐ray: The image demonstrates prominent interstitial markings suggestive of pulmonary edema.

**FIGURE 2:**
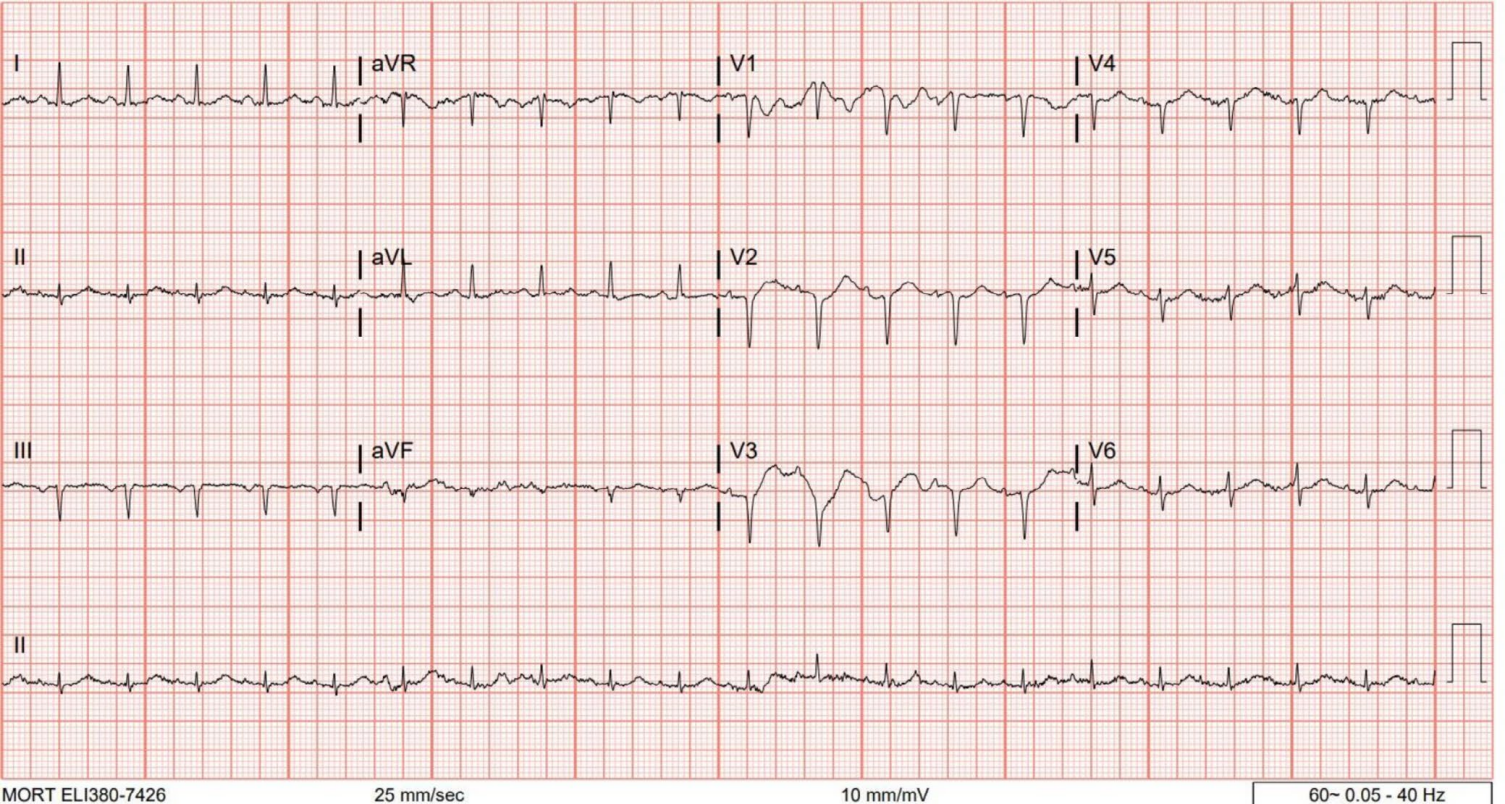
An electrocardiogram (ECG) on presentation showed sinus tachycardia with a normal axis, intervals, and ST‐T segments, but poor R‐wave progression and no acute ischemic changes.

Initial differential diagnosis included *β*‐blocker overdose, cardiogenic pulmonary edema, and ARDS. Labs: WBC 16,000/μL; bicarbonate 20 mmol/L; troponin 17 ng/L. CXR: pulmonary vascular congestion. The presenting chest radiograph demonstrated prominent interstitial markings suggestive of pulmonary edema (Figure 1). A representative admission ECG (Figure 2) demonstrated sinus tachycardia with poor R‐wave progression and no ischemic changes.

**FIGURE 3:**
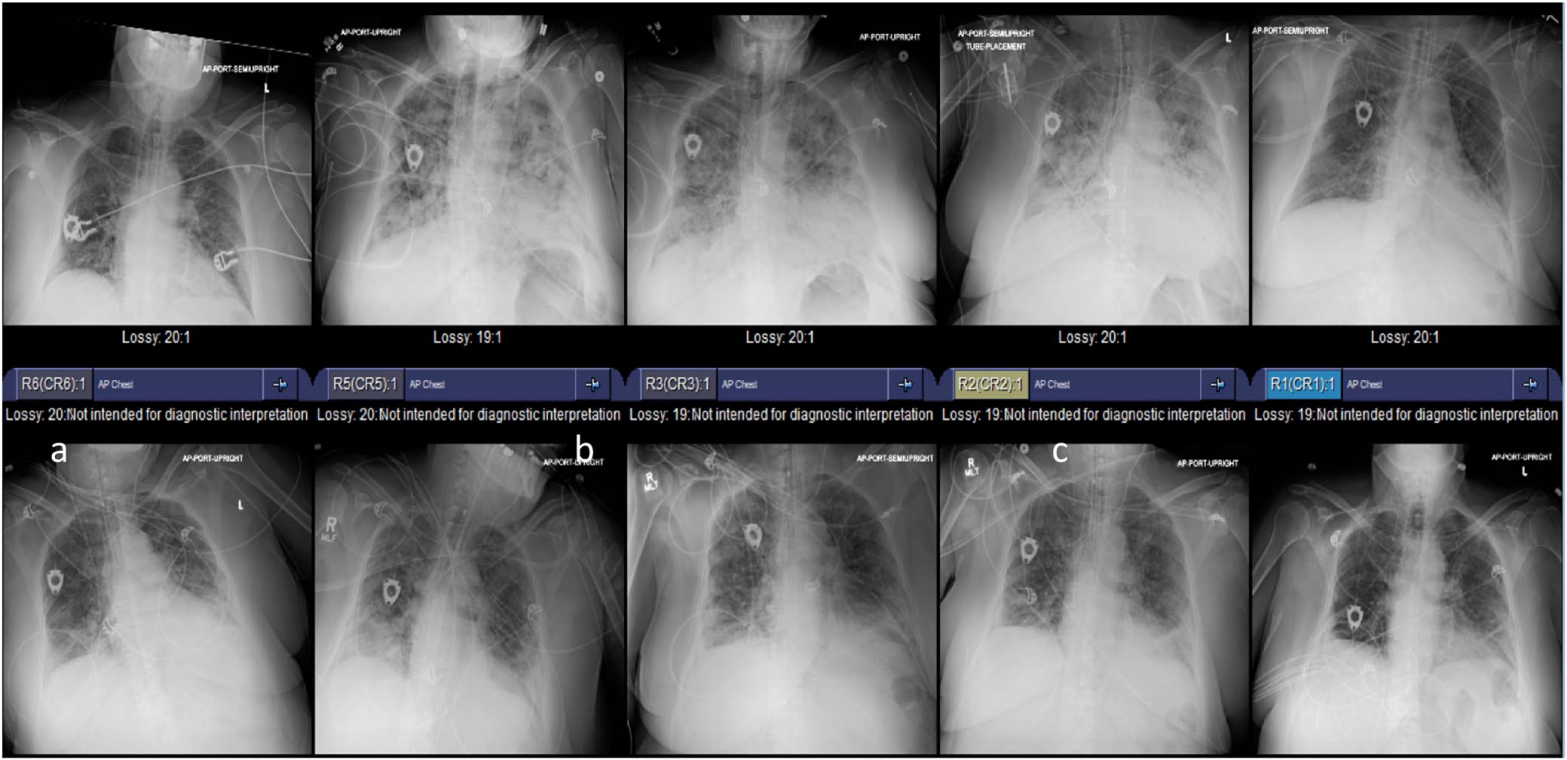
Serial chest radiographs demonstrating the evolution of pulmonary Edema: Sequential chest radiographs, arranged from the top left to the bottom right, depict the temporal progression of pulmonary interstitial edema. Initial images show worsening interstitial markings and vascular congestion, reaching maximal severity in the mid‐series, followed by gradual radiographic improvement consistent with resolving pulmonary edema.

Poison Control was contacted promptly and they recommended activated charcoal, whole bowel irrigation, serial glucose checks, fluid resuscitation, calcium gluconate, and vasopressors. Refractory hypotension required norepinephrine infusion. In the ICU, she received HIET, glucagon, and supportive therapy. Despite an elevated BNP (489 pg/mL) and mild troponin rise, echocardiography showed preserved LVEF (65%–70%), normal chamber dimensions, and no valvular abnormalities—including no aortic regurgitation—supporting NCPE rather than cardiogenic edema [5]. Serial chest radiographs showed progressive bilateral interstitial infiltrates followed by gradual radiographic improvement as pulmonary edema resolved (Figure 3).

She developed worsening hypoxemia and was subsequently intubated (SpO_2_ nadir 45%). Managed with high‐PEEP ventilation and 3 days of prone positioning. Objective ventilatory parameters (PaO_2_/FiO_2_ ratios and PEEP levels) are shown in Table 1 and satisfy Berlin ARDS criteria. By ICU day 4, she developed fever, leukocytosis (41.6 × 10^3^/μL), and worsening infiltrates consistent with VAP, which was treated with piperacillin–tazobactam. AKI, alkalosis, and electrolyte disturbances were also treated. She improved and was extubated on day 7. She completed a 7‐day antibiotic course and was discharged after 3 days on the medical floor with psychiatry follow‐up.

**TABLE 1:** Summary of arterial blood gas and ventilator parameters during the ICU stay. The table includes daily PaO_2_, FiO_2_, PaO_2_/FiO_2_ ratio, and PEEP values, along with clinical context, to illustrate the progression of oxygenation, severity of respiratory failure, and response to ventilatory strategies.

ICU day	PaO_2_ (mmHg)	FiO_2_ (%)	PaO_2_/FiO_2_ ratio	PEEP (cm H2O)	Clinical notes
Day 1	69	52%	133		HFNC; early hypoxemia
Day 2	65	80%	81		HFNC; worsening oxygenation
Day 3	60	100%	60	12	Intubated; severe ARDS
Day 4	45	100%	45	12	Prone positioning initiated
Day 5	69	40%	172	10–12	Improving oxygenation
Day 6	89	40%	222	10	Continued improvement
Day 7	84	60%	140	9	Weaning ventilation
Day 8	83	50%	166	9	Preparing for extubation
Day 9	76	50%	152	8	Extubated shortly after

### 2.3 Conclusion and Result

The patient developed acute respiratory failure with NCPE following a massive amlodipine overdose. Her preserved EF and lack of valvular disease strongly supported NCPE rather than cardiogenic pulmonary edema. Aggressive ICU management resulted in full recovery.

## 3 Discussion

Calcium channel blockers (CCBs) such as amlodipine are widely used for the treatment of hypertension and angina. While effective in managing cardiovascular conditions, these drugs pose a significant risk when ingested in overdose, as they can cause severe hypotension, bradycardia, and even death [1]. This case underscores the clinical challenges associated with CCB toxicity, emphasizing the need for prompt recognition, aggressive intervention, and careful monitoring of both hemodynamic status and laboratory parameters.

Amlodipine, a dihydropyridine calcium channel blocker, primarily acts on vascular smooth muscle, leading to vasodilation and a reduction in systemic vascular resistance [1, 6]. In overdose, the resulting excessive vasodilation and myocardial depression can lead to severe hypotension, impaired cardiac output, and organ hypoperfusion [1, 6, 8] Additionally, amlodipine can impair insulin secretion and cause hyperglycemia, which compounds the effects of overdose [2]. The presence of hyperglycemia in a nondiabetic patient is a recognized distinguishing feature of CCB toxicity when differentiating calcium channel blocker (CCB) poisoning from beta‐blocker toxicity in cases of diagnostic uncertainty [2, 3, 7].

The patient presented with signs and symptoms of a large CCB overdose, including hypotension, tachycardia, and elevated troponin levels. Troponin elevation in CCB toxicity is likely due to myocardial injury from prolonged hypotension and impaired oxygen delivery [1]. Chest X‐ray showed pulmonary congestion, and the patient developed noncardiogenic pulmonary edema (NCPE), reflecting systemic effects of the overdose worsened by sustained hypotension. The diagnosis of NCPE was supported by preserved LVEF, absence of valvular or regional wall‐motion abnormalities, and radiographic findings consistent with ARDS physiology [5].

To further differentiate cardiogenic vs. noncardiogenic pulmonary edema, we evaluated objective oxygenation parameters. The patient's PaO_2_/FiO_2_ ratios, need for high PEEP, and improvement with prone positioning as shown in Table 1 were characteristic of ARDS/NCPE rather than hydrostatic edema [5]. Although BNP was elevated, this value is nonspecific in critical illness and does not exclude NCPE when cardiac function is preserved.

**FIGURE 4:**
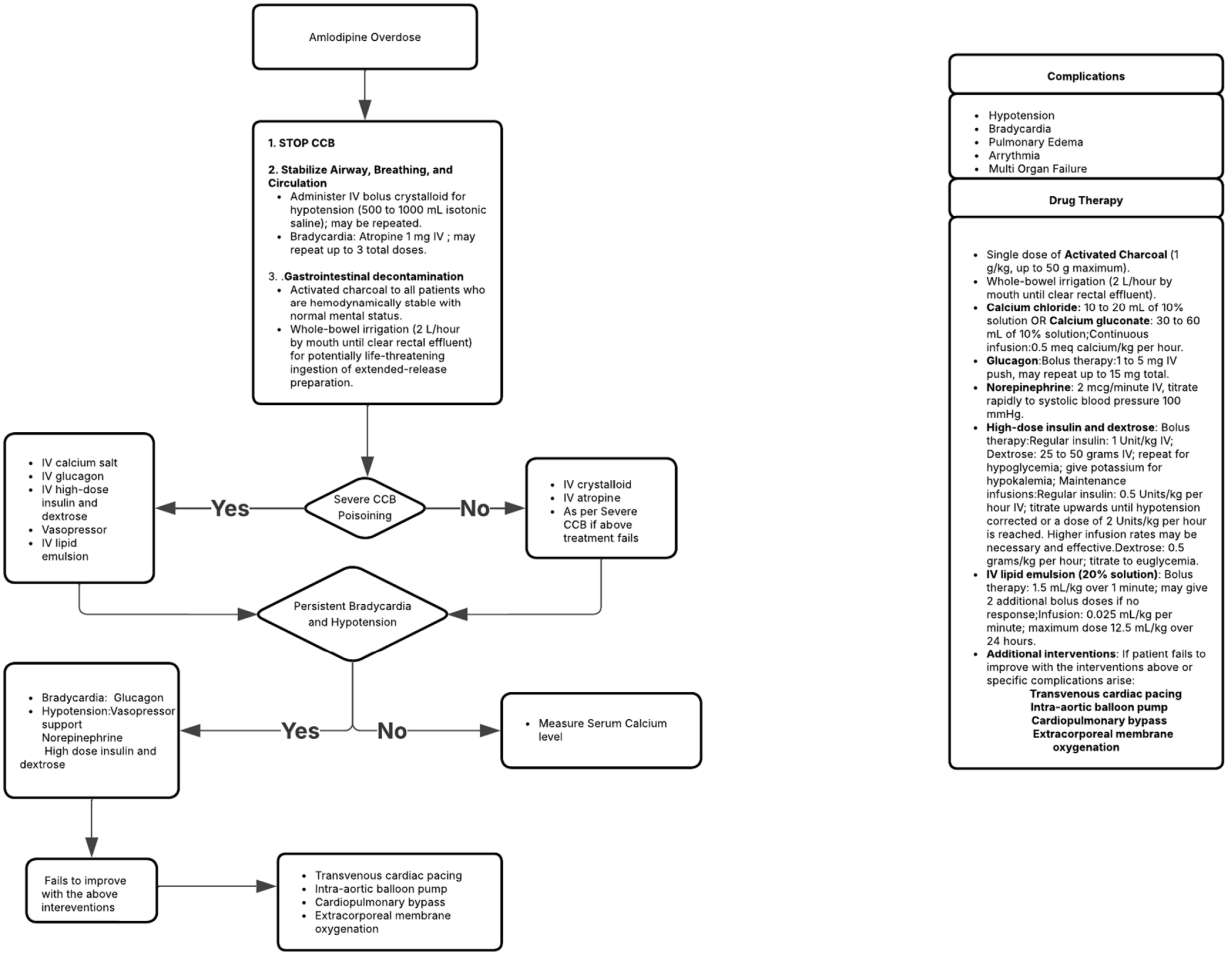
Schematic representation of an overview of the clinical management approach (8). Severe CCB poisoning is characterized by significant hemodynamic compromise and metabolic disturbances, which include profound hypotension, bradycardia, hyperglycemia, altered mental status, metabolic acidosis, and cardiogenic shock.

The management of calcium channel blocker (CCB) overdose (Figure 4) involves supportive care along with specific antidote therapies [9]. The cornerstone of treatment is aggressive IV fluid resuscitation to correct hypotension and improve organ perfusion. In this patient, the administration of IV fluids and vasopressors, particularly norepinephrine, was critical in maintaining hemodynamic stability. Norepinephrine is recommended as the first‐line vasopressor for CCB‐induced vasodilatory shock, as outlined by the international expert consensus recommendations for calcium channel blocker poisoning [7, 8, 10].

In cases of severe calcium channel blocker (CCB) toxicity, interventions like glucagon and hyperinsulinemic euglycemic therapy (HIET) have shown promise [3, 4, 6, 7, 8]. HIET involves high‐dose insulin and glucose infusion, provides an energy substrate for the heart, and reverses negative inotropic effects of CCBs [4]. HIET is considered a first‐line therapy in severe CCB overdose with shock, especially when vasopressors alone are insufficient [3, 4, 7, 8].

The development of NCPE in this patient highlights a serious complication of CCB overdose. The pathophysiology is thought to result from systemic vasodilation and endothelial dysfunction [5, 6]. The absence of significant cardiac pathology supported NCPE rather than cardiogenic pulmonary edema. Sepsis‐induced ARDS was considered after fever, leukocytosis, and new infiltrates appeared; however, these signs developed after the onset of pulmonary edema. Thus, VAP may have prolonged the respiratory course, but the initial presentation reflected NCPE due to amlodipine toxicity.

Management of NCPE requires optimizing oxygenation and ventilation while treating the underlying cause. Intubation, lung‐protective ventilation, and sustained proning were essential. Electrolyte abnormalities were managed aggressively. The patient met Berlin ARDS criteria, further supporting NCPE over cardiogenic pulmonary edema.

ECMO has been used in refractory cases [11, 12], but was not required due to improvement with medical therapy. In prior reports, young patients survived ingestion of 900 mg amlodipine without major comorbidities [13, 14]. Our patient survived 870 mg despite significant comorbidities, demonstrating the effectiveness of timely HIET, norepinephrine, and ARDS ventilation strategies [13, 14].

This case reinforces key clinical principles.

[1] NCPE should be considered even when BNP is elevated.

[2] Echocardiography and ARDS criteria are essential to differentiate NCPE from cardiogenic edema.

[3] HIET and norepinephrine are cornerstone therapies.

[4] Early lung‐protective ventilation including proning can significantly improve outcomes.

## 4 Conclusion

This case illustrates the complexities involved in the management of CCB overdose, particularly in a patient with significant comorbidities. Effective management requires a multidisciplinary approach, including supportive care, pharmacologic therapy, and careful monitoring for complications such as NCPE and electrolyte abnormalities. Systematic reporting of similar cases may help refine diagnostic criteria and management strategies for NCPE in amlodipine toxicity [5, 13, 14].

## Author Contributions

**Rohit Pandit:** conceptualization, supervision, writing – original draft, writing – review and editing. **Pradeep Masuta:** conceptualization, supervision, writing – review and editing. **Nishchal Regmi:** conceptualization, writing – original draft, writing – review and editing. **Anil Nepali:** conceptualization, writing – original draft, writing – review and editing. **Gajendra Acharya:** supervision, writing – review and editing. **Muhammad Jibran:** conceptualization, writing – review and editing. **Heena Maharjan:** writing – original draft, writing – review and editing. **Nimesh Shrestha:** writing – original draft.

## Funding

The authors have nothing to report.

## Ethics Statement

The authors have nothing to report.

## Consent

Written informed consent was obtained.

## Conflicts of Interest

The authors declare no conflicts of interest.

## Data Availability

Available upon reasonable request.
